# Metacontrol-related aperiodic and periodic neural activity in cognitive aging: enhancing the neural signal-to-noise ratio through anodal transcranial direct current stimulation

**DOI:** 10.1007/s11357-025-02077-8

**Published:** 2026-01-16

**Authors:** Yu Pi, Qinfei Zhang, Shuhui Lyu, Christian Beste, Lorenza Colzato, Bernhard Hommel

**Affiliations:** 1 Shandong Provincial Key Laboratory of Brain Science and Mental Health, Faculty of Psychology, Shandong Normal University, Jinan, China No. 88 East Wenhua Road, Shandong Province 250014; 2 Cognitive Neurophysiology, Department of Child and Adolescent Psychiatry, Faculty of Medicine, TU Dresden, Dresden, Germany

**Keywords:** Aperiodic activity, Theta oscillations, Metacontrol, Cognitive control, Aging, TDCS

## Abstract

Metacontrol, the ability to adapt cognitive control to task demands, declines with age and is thought to be reflected in aperiodic and periodic neural dynamics. Given that anodal transcranial direct current stimulation (atDCS) can modulate cortical excitability via membrane potential shifts, we tested whether atDCS alters the neurophysiological signatures of metacontrol in younger and older adults. In a mixed design, younger and older participants performed a Go/Nogo task under both atDCS and sham stimulation conditions; resting-state EEG data were also acquired. Aperiodic activity was analyzed using the FOOOF algorithm, and periodic activity was examined through time–frequency analysis. Behaviorally, younger adults showed higher accuracy and faster responses than older adults, but no significant stimulation effects emerged in either group. Results showed that, compared to sham, aperiodic activity (FOOOF exponent) increased after atDCS, particularly in older adults, indicating a steepening of the EEG spectrum and thus increased inhibitory tone in the aging process. However, resting-state aperiodic activity did not predict stimulation-induced effects within either group. In the periodic domain, we found no evidence that atDCS modulated task-related theta or alpha power. Moreover, exploratory analyses revealed no significant associations between atDCS-induced changes in the aperiodic exponent and oscillatory power. This dissociation indicates that, under the present conditions, the periodic and aperiodic components of the EEG signal reflect distinct and likely independent neurophysiological responses to neuromodulation. Targeting metacontrol mechanisms through neuromodulation may, with further validation, open new avenues for supporting cognitive health in older adults.

**Graphical Abstract:**

**Figure:**
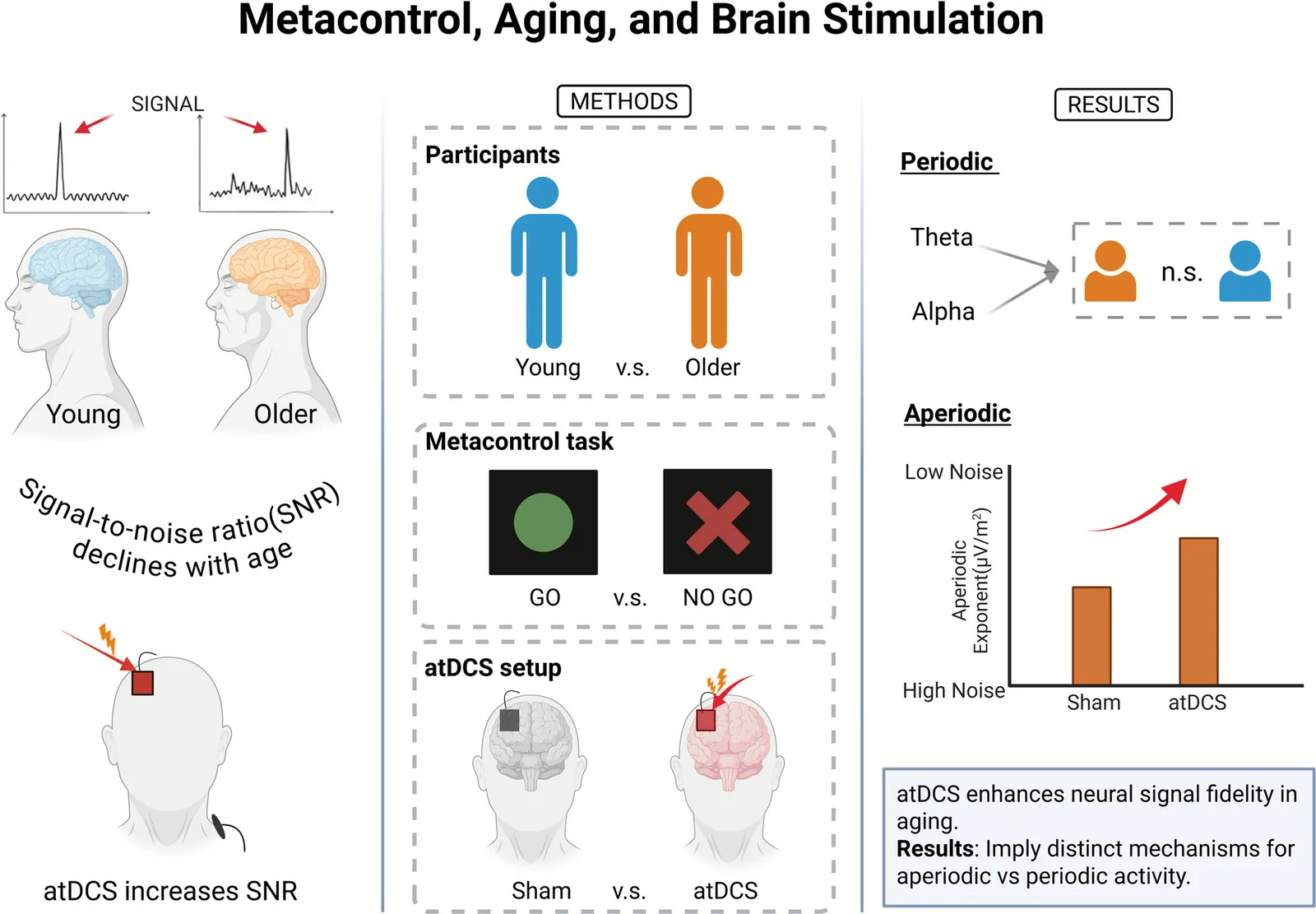

**Supplementary Information:**

The online version contains supplementary material available at https://doi.org/10.1007/s11357-025-02077-8.

## Introduction

Aging is associated with progressive declines in physiological health and cognitive functioning, a phenomenon of increasing societal concern given the growing proportion of older adults worldwide [1–4]. Among cognitive domains, cognitive control—the set of processes enabling goal-directed thought and adaptive behavior—declines substantially with age [2–4]. Traditional models have emphasized self-regulatory functions, such as maintaining goal-directed focus and suppressing distractions [5–7]. However, emerging frameworks suggest that adaptive control extends beyond mere persistence: effective behavior often requires balancing persistence with flexibility, allowing disengagement from fixed goals and receptivity to new information [8–12]. This dynamic regulation of cognitive control has been conceptualized as metacontrol, reflecting the capacity to flexibly adjust the balance between persistence and flexibility depending on context [9–12].

Metacontrol is particularly vulnerable to aging. Control processes reach peak efficiency in early adulthood but decline with advancing age, largely due to structural and functional changes in frontal regions and alterations in key neurotransmitter systems, including dopamine and gamma-aminobutyric acid (GABA) [13–16]. GABA, the brain’s primary inhibitory neurotransmitter, decreases with age and is crucial for maintaining the excitation/inhibition (E/I) balance that supports stable and efficient neural processing [17–19]. Age-related reductions in GABA contribute to altered cortical excitability and impaired cognitive control, particularly in prefrontal regions implicated in metacontrol [19].

Neural activity can be broadly characterized as periodic—reflecting oscillatory rhythms such as theta and alpha bands—or aperiodic, representing scale-free, broadband fluctuations in EEG power. Interestingly, aperiodic activity provides a sensitive index of metacontrol [17–23]. Conceptually, a higher aperiodic exponent reflects a steeper slope of the EEG power spectrum in log–log space, indicating stronger inhibitory regulation and more stable neural dynamics. Conversely, a lower exponent denotes relatively less inhibitory regulation [24–26]. Critically, while raw FOOOF exponent values can be positive or negative, an increase in the exponent (toward less negative or more positive values) corresponds to a steepening of the power spectrum, which is associated with increased inhibitory tone supporting cognitive persistence [17–23]. Complementing this, periodic activity in theta and alpha bands provides additional insight into cognitive control: theta rhythms are associated with active engagement and top-down control, whereas alpha rhythms reflect inhibitory gating of irrelevant information, both of which decline with age [27, 28]. Together, these measures offer complementary perspectives on the neural mechanisms supporting persistence and flexibility in adaptive behavior.

Noninvasive brain stimulation offers a promising avenue for modulating these age-related changes. Several magnetic resonance spectroscopy (MRS) and neurophysiology studies converge in showing that, although anodal transcranial direct current stimulation (atDCS) is classically described as increasing cortical excitability via subthreshold neuronal depolarization, its net systems-level effect also involves modulation of GABAergic inhibitory tone. Bachtiar et al. [29] and Antonenko et al. [30] demonstrated that anodal tDCS can functionally reorganize GABA levels in a stimulation-site- and age-dependent manner, accompanied by changes in resting-state network connectivity. Inoue et al. [31] further showed that the direction and magnitude of GABA modulation depend on montage and neurophysiological state, suggesting targeted enhancement of inhibitory interneuron efficacy rather than simple excitation. Overall, these findings indicate that anodal tDCS does not merely “excite” the cortex but can fine-tune the E/I balance via modulation of GABAergic systems and related strengthening of inhibitory regulation.

Building upon this framework, the present study investigated whether atDCS can modulate metacontrol in aging by altering both aperiodic and periodic neural dynamics. Two primary hypotheses guided this work. First, we predicted that atDCS would increase aperiodic exponents of EEG activity, reflecting stronger inhibitory regulation, particularly under conditions requiring high cognitive persistence. We further anticipated that these effects would be state-dependent, emerging primarily during active task engagement when inhibitory demands are high, because the stimulation would reinforce the neural circuits underlying the adaptive expression of persistence in metacontrol.

Second, we hypothesized that individual differences in baseline aperiodic activity—measured at rest—would predict responsiveness to atDCS, with individuals exhibiting lower baseline exponents (reflecting relatively weaker inhibitory regulation) benefitting more from stimulation. Additionally, we explored potential interactions between aperiodic and oscillatory (theta/alpha) activity to provide a comprehensive account of the neural mechanisms underlying persistence and flexibility in aging.

## Methods

### Participants

We calculated the required minimal sample size (of 30 participants per group) using G*Power (version 3.1.9.2), specifying an alpha level of 0.05, a power of 0.90, and an expected effect size of *f* = 0.25. The study included N = 40 healthy right-handed younger participants (age range: 18 ~ 30, M ± SD_age_ = 22.35 ± 3.81; 22 women) and N = 40 healthy right-handed older participants (age range: 55 ~ 65, M ± SD_age_ = 61.28 ± 3.00; 24 women) recruited in Lixia, the metropolitan area of Jinan, where the Shandong Normal University is located. Due to insufficient EEG data quality, four younger and one older participant were excluded, resulting in a final sample of N = 36 younger adults (age range: 18 ~ 30, M ± SD_age_ = 21.78 ± 3.26; 18 women) and N = 39 older adults (age range: 55 ~ 65, M ± SD_age_ = 61.18 ± 2.97; 24 women) for data analysis. All participants had normal or corrected-to-normal vision and were screened prior to enrollment through a detailed health questionnaire and a verbal interview, and met the safety standards required for atDCS stimulation [5–7]. Inclusion criteria consisted of being in the respective age group for young or older adults and right-handedness. Exclusion criteria encompassed any major neurological or psychiatric disorder, any unstable or uncontrolled cardiovascular condition (e.g., hypertension, coronary heart disease and myocardial infarction), contraindications for tDCS such as implantable medical devices or metal in the head region, current pregnancy or lactation, and regular use of psychoactive medications affecting cortical excitability. Importantly, all participants were first-time participants in an atDCS study and had not engaged in similar studies with brain stimulation devices previously. Before the first session, participants received written instructions detailing the study procedures, and all provided written informed consent. Upon completion of both testing sessions, each participant was compensated with 240 RMB. The study adhered to the Declaration of Helsinki and was approved by the School of Psychology's Ethics Committee at Shandong Normal University.

### Research design and methodology

The study was conducted in one of the EEG labs at the Metacontrol lab of the School of Psychology at Shandong Normal University in China. A within-participant, single-blind, crossover design was employed, wherein participants underwent both active anodal tDCS (atDCS) and sham stimulation conditions in separate sessions. The order of sessions (atDCS first vs. sham first) was counterbalanced and randomized across participants, with the two testing sessions spaced seven days apart to minimize carryover effects. The participants were blinded to the stimulation condition, while the experimenter administering the stimulation was necessarily aware of it due to the equipment setup. To minimize potential experimenter bias, all task instructions and procedures following stimulation were strictly standardized and scripted. Each session began with a 6-min resting-state EEG recording, which was conducted under an eyes-open condition (for detailed see Sect. "Resting-state EEG" below). This was immediately followed by a 20-min period of either real or sham atDCS administered immediately before the cognitive task. The preparation and waiting times were identical for both conditions. To formally assess the effectiveness of participant blinding, all participants completed a tDCS Safety Questionnaire at the end of each session. This questionnaire specifically inquired about the sensory experiences (e.g., pain, tingling, itching, or burning under the electrodes) during and after stimulation. An independent samples t-test comparing pain sensation reports between real and sham conditions revealed no significant difference (*t* = 0.33, *p* = 0.743), confirming that participants could not reliably distinguish between the two stimulation conditions based on their sensory experiences.

### Resting-state EEG

Participants' open-eye resting EEG activity was recorded for 6 min. Throughout the recording, participants were instructed to keep their eyes open, blink normally, and fixate on a white central crosshair presented against a black background. The EEG was acquired using 64 isometrically positioned Ag/AgCl electrodes and amplified by a BrainAmp amplifier (Brain Products GmbH, Gilching, Germany). The ground electrode was placed at the AFz position, and the online reference electrode was placed at FCz. The sampling frequency was set at 500 Hz, and electrode impedances were maintained below 5 kΩ.

### Offline atDCS stimulation

**Fig. 1:**
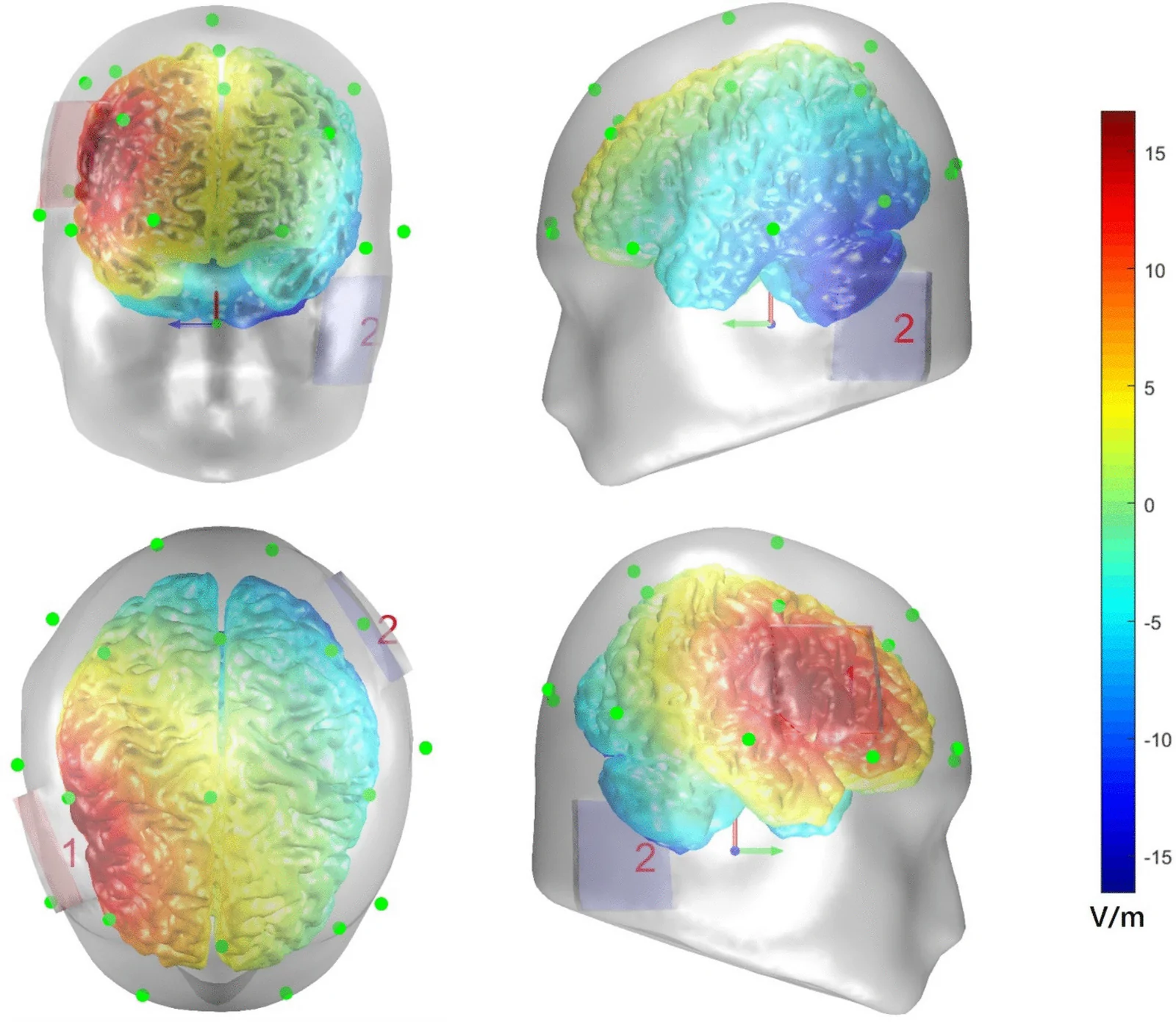
Visual representation of the electrode arrangement used for tDCS: 1) Anode; 2) Reference electrode (cathode). A color-bar simulation of the resulting voltage flow, calculated using the COMETS2 toolbox in Matlab, is displayed. The color bar indicates the electric field strength in V/m

To non-invasively modulate cortical excitability within the frontal-parietal network, atDCS was applied over the rIFG. To justify and optimize our stimulation montage, we first performed electric field modeling using the "COMETS2" toolbox for MATLAB [8]. The model predicted that placing the anode at the midpoint between electrodes FC4 and F8, with the cathode placed contralaterally between the neck and deltoid muscle, would effectively generate an electric field focused on the target rIFG (see Fig. 1). According to previous research [9–11], participants received either 20 min of anodal stimulation or sham stimulation targeting the rIFG. Anodal stimulation was administered using a NeuroConn DC-Stimulator Plus device (NeuroConn, Ilmenau, Germany) through two rubber electrodes (5 × 5 cm^2^, NeuroConn, Ilmenau, Germany). A Ten20 conductive paste (0.5 mm thick, shaped with custom casing; [12] was utilized to maintain impedances below 5 kΩ. To minimize commonly reported unpleasant sensations, the current fade-in and fade-out times were set to 15 s each. In the real stimulation condition, a 2-mA current was applied for 1200 s (20 min), with 15-s fade-in and fade-out periods, prior to participants performing the intensity-modulated Go-Nogo task. In the sham stimulation condition, the 2-mA current was applied for only 30 s, with a 15-s fade-in and fade-out, to mimic the initial tingling sensation typical of true non-invasive brain stimulation.

### Task

**Fig. 2:**
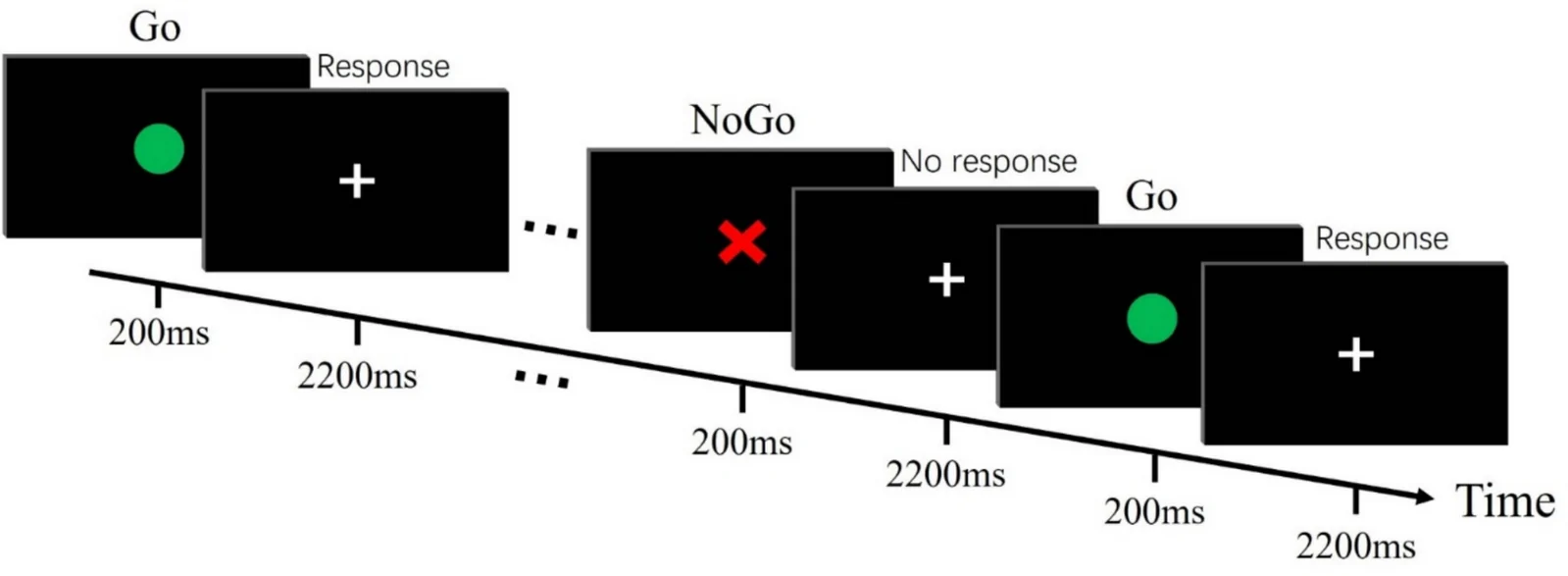
The Go-Nogo paradigm. The task consists of 70% Go trials and 30% Nogo trials. The Go and Nogo stimuli are presented for 200 ms, followed by a response window of 2200 ms. During Go trials, participants are required to make a quick key response when the Go stimulus is presented. Conversely, during Nogo trials, participants are instructed to refrain from making any keystroke response when the Nogo stimulus is presented

A Go-Nogo task programmed using "Presentation" software package (Neurobehavioural Systems; https://www.neurobs.com/) was employed. As illustrated in Fig. 2, this task comprised a distribution of 70% Go trials and 30% Nogo trials, designed to establish a pre-potent response execution tendency, thereby increasing the challenge of inhibiting responses during Nogo trials. Participants were instructed to react or refrain from reacting to specific visual stimuli as swiftly as possible. The stimuli consisted of a "  " for Go trials and a "  " for Nogo trials, displayed on a 21-inch computer screen. In Go trials, participants were required to press a button with their right hand within 2200 ms post-stimulus presentation; failure to respond was recorded as a no-hit. Conversely, in Nogo trials, any response within 2200 ms was documented as a false alarm, indicating an executed response. Each trial lasted 2400 ms for Go trials and Nogo trials, with the picture stimulus presented for 200 ms. The experiment comprised 400 trials, which included 280 Go trials and 120 Nogo trials, evenly divided into four blocks of 100 trials each, with an equal distribution of trial types across all blocks. The sequence of trials was randomized within each block.

### EEG recording and analysis

The data were analyzed offline using Analyzer 3.0, both the resting-state data and the data from the Go/Nogo task underwent initial downsampling to 256 Hz. Flat channels were then removed, and the EEG data were re-referenced to an average reference. A 0.5 to 50 Hz IIR band-pass filter (Order 8), along with a 50 Hz notch filter, was applied to both the resting-state and task-related data. The raw data were manually inspected to eliminate irregular artifacts. Subsequently, Independent Component Analysis (ICA) using the Infomax restricted algorithm was employed to identify and manually remove recurrent artifacts such as horizontal and vertical eye movements, blinks, and pulses. Following ICA, a semi-automatic manual inspection was conducted to remove any remaining unidentified artifacts.

The resting-state data were segmented into 1000 ms intervals starting from the onset of the resting-state stimulus. Task-related data were segmented and aligned with the onset of Go and NoGo stimuli, with each segment lasting 4000 ms, spanning from 2000 ms before to 2000 ms after stimulus onset. Further analysis focused on Go trials where participant responses occurred 2200 ms after stimulus onset, and Nogo trials where no response was given within 2200 ms post-stimulus onset. Segments exhibiting amplitudes below −100 µV or above 100 µV, or with activity below 0.5 μV within a 100 ms interval, were automatically rejected. Finally, baseline correction was conducted on the task-related EEG data from −200 ms to 0 ms (stimulus onset), and segments were averaged for each participant in both Go and Nogo conditions.

### Parameterization of the spectral data

During the resting-state measurement, the continuous EEG data were segmented into 1000 ms epochs, each treated as a pseudo-trial time-locked to the beginning of the segment (0–1000 ms). This duration was selected to ensure segment lengths comparable to those derived from event-related experimental data, presuming a similar signal-to-noise ratio. In the context of the Go-Nogo task, the interval from 1000 ms prior to stimulus onset to 0 ms was designated as the pre-trial period, while the period from 0 to 1000 ms following stimulus onset was identified as the within-trial period.

To minimize contamination from transient, time-locked evoked potentials such as the visual evoked potential (VEP) and movement-related cortical potentials in the task data, we adopted the following procedure: the event-related potential (ERP) was first computed by averaging the time-domain segments within each condition for each participant. This ERP, containing the phase-locked activity, was then subtracted from each individual trial. Subsequent spectral analysis was performed on these residual, non-phase-locked data segments to isolate the oscillatory and aperiodic components of interest.

The power spectral density (PSD) for both the resting-state measurement and the pre-trial/within-trial periods of the Go/Nogo task was estimated using Welch’s method, implemented via the “welch” function in MATLAB. For the 1000 ms epochs, we used a 250 ms Hanning window with 50% overlap [13]. This configuration provides a frequency resolution of 1 Hz (as determined by the reciprocal of the full epoch length, 1/1 s = 1 Hz), allowing for reliable estimation within the 4–40 Hz range of interest. To obtain robust spectral estimates for parameterization, the PSDs from all individual trials within each experimental condition were first averaged for each participant. The resulting mean power spectra were then parameterized using the Python-based FOOOF toolbox (version 1.0.0; accessible at https://github.com/fooof-tools/fooof) to quantify aperiodic activity [14]. In brief, FOOOF decomposes the power spectrum into aperiodic (1/f) and periodic (oscillatory) components. The aperiodic component is characterized by the exponent, which reflects the slope of the spectrum. The full model equation, fitting procedures, and parameters (e.g., peak width limits, minimum peak height) are detailed in the Supplementary Material S1.1. The model was fitted to the 3–40 Hz range for each electrode, participant, and condition. The average goodness-of-fit (R^2^) across the 75 participants was 0.92, indicating good model performance.

### Aperiodic exponent

The aperiodic parameters encompass both the aperiodic exponent and aperiodic offset. Previous studies have demonstrated that the aperiodic exponent reflects sensitivity to the metacontrol state index [15–19], our analysis focused primarily on this exponent. Aperiodic exponents were analyzed during the pre-stimulus interval (pre-trial period), when participants remained unaware of the forthcoming persistence demands, and thus no differentiation between persistence-demanding and -undemanding conditions was expected. These measures were then compared to aperiodic exponents recorded during the post-stimulus interval (within-trial period), when participants had acquired awareness of the condition-specific control demands. A predominant or exclusive modulation of aperiodic exponents in the within-trial phase by atDCS would be indicative of a highly task-specific effect. For each participant and electrode, the aperiodic exponent was derived. Given the absence of strong a priori hypotheses regarding the precise spatial focus of stimulation or group effects, we employed a two-pronged analytical approach. First, following the "global" exponent approach recommended by [32], we averaged the aperiodic exponent values across all 64 electrodes for each participant to obtain a single, whole-scalp summary metric for use in group-level statistical comparisons. Subsequently, to complement this global analysis and explore whether there were any condition-specific differences (e.g., between age groups or stimulation conditions) that exhibited a spatially structured pattern across the scalp, we performed non-parametric cluster-based permutation tests using the MNE-Python toolbox (version 1.5.1; accessible at https://mne.tools/stable/index.html) [33]. This data-driven method identifies contiguous clusters of electrodes that show a significant effect, while automatically correcting for multiple comparisons across the scalp. The analysis was conducted by comparing the full spatial distribution of exponent values between conditions (e.g., Young vs. Older adults). Clusters were defined based on spatial adjacency of thresholded sample-level F-values (α = 0.05), with cumulative cluster-level F-values serving as the test statistic. Significance was determined using 1,000 Monte Carlo permutations and a cluster-defining threshold of *p* < 0.05. A detailed description of the time–frequency analysis procedures is provided in the Supplementary Material S1.2.

### Statistical analysis

All statistical analyses were performed using SPSS software (IBM, version 27.0). Behavioral data were analyzed using 2 (Stimulation: atDCS vs. sham) × 2 (Group: young vs. old) mixed-design ANOVA to examine the effects of stimulation and age on hit rates and reaction times (RTs) during Go trials, as well as false alarm rates during Nogo trials.

For neurophysiological measures, to test the first hypothesis that atDCS would reduce cortical neural noise, analyses of aperiodic activity were conducted using a whole-brain four-way repeated-measures ANOVA with one between-subject factor (Group: younger vs. older) and three within-subject factors: time window (pre-trial vs. within-trial), stimulation session (atDCS vs. sham), and task condition (Go vs. Nogo). Following this, separate three-way repeated-measures ANOVAs were performed for each time window (pre-trial and within-trial) with factors Group × Stimulation × Go/Nogo. To further explore the stimulation effect within the within-trial time window, two-way repeated-measures ANOVAs (Stimulation × Go/Nogo) were conducted separately for each age group. This hierarchical approach allowed us to investigate whether the hypothesized modulation of nonperiodic activity by atDCS differed between younger and older participants.

To further examine individual variability in stimulation-induced neuromodulation, we computed the ΔatDCS value (i.e., the difference between atDCS and sham conditions in aperiodic activity). The resting-state aperiodic exponent used in the subsequent correlation analysis was computed as the average of the two eyes-open (EO) resting-state recordings collected at the beginning of both the atDCS and sham sessions, providing a stable baseline measure for each participant. A Pearson correlation analysis was then conducted between this averaged resting-state aperiodic exponent and ΔatDCS to test the second hypothesis that baseline aperiodic activity levels predict the degree of neuromodulatory response to atDCS in each age group. Exploratory analyses examining time–frequency dynamics and correlations between stimulation-induced changes in aperiodic activity and oscillatory dynamics are described in the Supplementary Material S1.3. The Greenhouse–Geisser correction was applied to all tests, and further simple effects analyses were conducted only when interactions were significant. All post hoc tests were corrected using the Bonferroni method. Descriptive statistics were reported as the mean and standard error of the mean (SEM).

## Results

### Behavior

For the Go condition, the analysis of hit rate revealed a significant main effect of age group, *F*_[1, 73]_ = 8.76, *p* = 0.004, η^2^ₚ = 0.10, with younger adults exhibiting higher accuracy than older adults. Similarly, the RTs for Go trials showed a significant main effect of age, *F*_[1, 73]_ = 21.43, *p* < 0.001, η^2^ₚ = 0.21, with faster responses in the younger group. No significant main effect of stimulation or interaction effect was found for either hit rate (*F*s < 1.5, *ps* > 0.2) or RTs (*F*s < 1.0, *ps* > 0.3). In the Nogo condition, the analysis of false alarms showed no significant effects of age, stimulation, or their interaction (*Fs* < 1.2, *ps* > 0.2).

### PSD

**Fig. 3:**
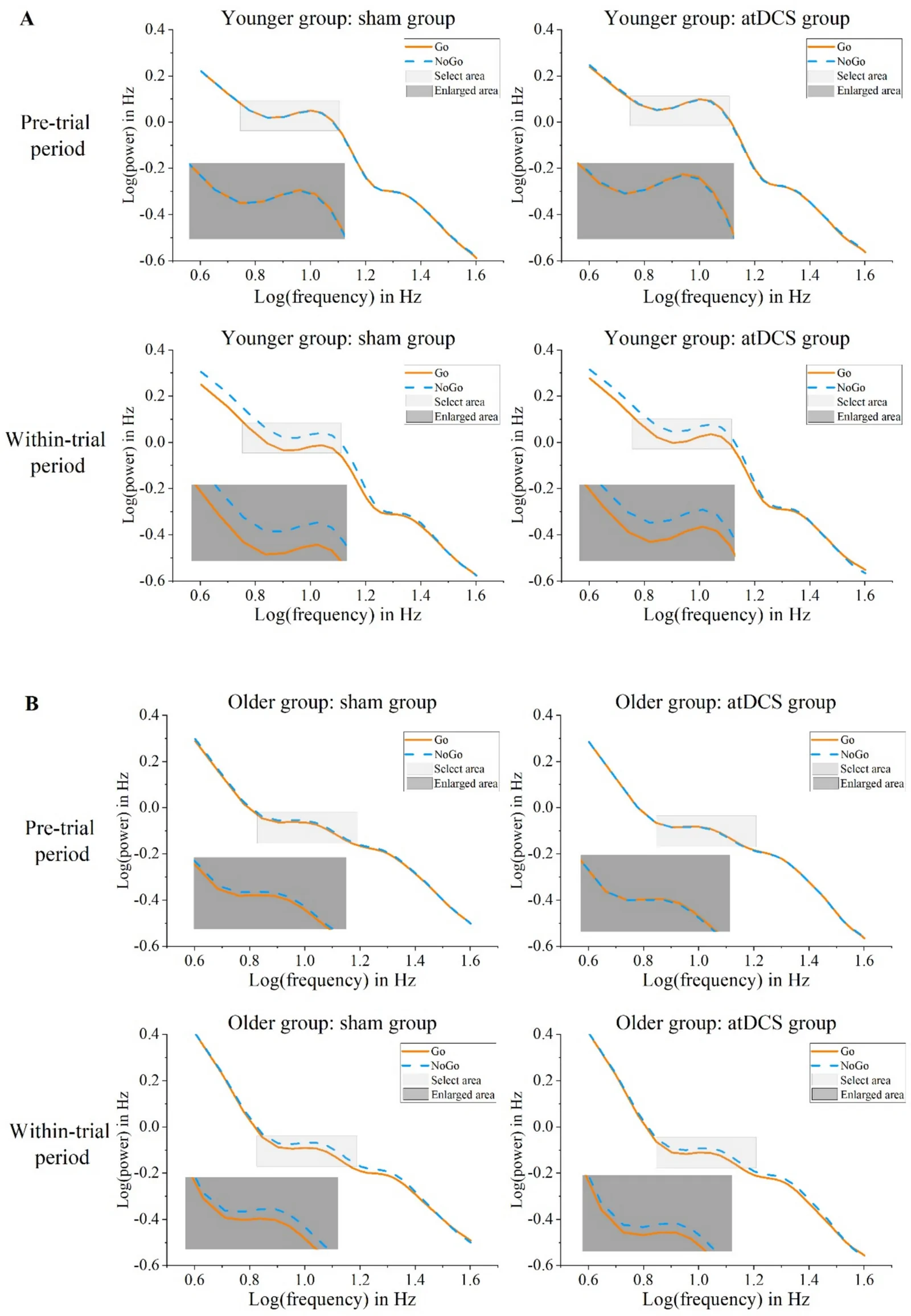
The log–log transformed power spectral density plot presents averaged data across all electrodes and participants. (**A**) show the PSDs of the young group for the sham and atDCS stimulations in the pre-trial and within-trial periods; (**B**) show the PSDs of the older group for the sham and atDCS stimulations in the pre-trial and within-trial periods

Figure 3 illustrates the PSDs in log–log space for younger and older groups across the 4–40 Hz frequency range, comparing atDCS and sham stimulation during pre-trial and within-trial periods. These PSDs were calculated by averaging over all electrodes and participants.

### Aperiodic exponent (brain-wide): analysis to test Hypothesis 1

A whole-brain four-way repeated-measures ANOVA (Time Window × Stimulation × Task Condition × Age Group) revealed several key findings. First, a significant main effect of time window emerged (*F*_[1,73]_ = 105.45, *p* < 0.001, η2 p = 0.59, 95% CI [0.025, 0.038]), indicating an increased aperiodic exponent during the within-trial period (0.98 μV/m^2^ ± 0.04) compared to the pre-trial period (0.95 μV/m^2^ ± 0.04). Additionally, main effects were observed for age group (*F*_[1,73]_ = 6.80, *p* = 0.011, η2 p = 0.09, 95% CI [0.048, 0.362]), with younger adults (1.07 μV/m^2^ ± 0.06) showing generally higher aperiodic exponents than older adults (0.87 μV/m^2^ ± 0.05), and for task condition (*F*_[1,73]_ = 26.70, *p* < 0.001, η2 p = 0.27, 95% CI [0.011, 0.033]), with higher exponents in Nogo trials (0.98 μV/m^2^ ± 0.04) than Go trials (0.96 μV/m^2^ ± 0.04). Significant two-way interactions were also found between time window and task condition (*F*_[1,73]_ = 125.03, *p* < 0.001, η2 p = 0.63). Most importantly, a significant three-way interaction of time window × age group × task condition was observed (*F*_[1,73]_ = 30.54, *p* < 0.001, η2 p = 0.30), indicating that age-related differences in aperiodic dynamics were modulated by task demands during specific temporal phases.

**Fig. 4:**
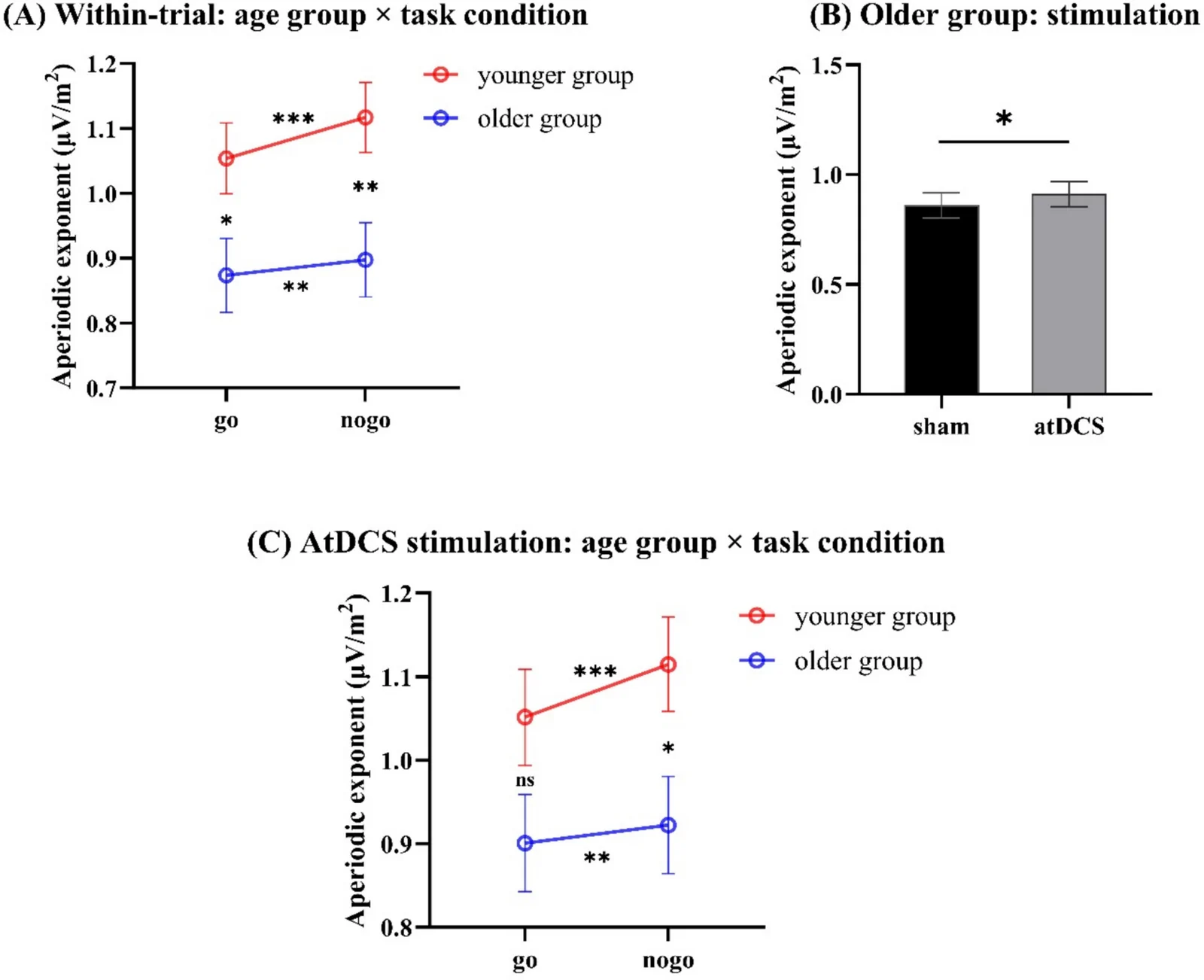
(**A**) Aperiodic exponents for younger and older adults during Go and Nogo trials in the within-trial period; (**B**) Aperiodic exponents for sham and atDCS stimulation conditions in older adults during the within-trial period; (**C**) Aperiodic exponents for younger and older adults during Go and Nogo trials under atDCS stimulation in the within-trial period. The line plots display the mean value of the corresponding condition, and the error bars display the SEM. ^*^ *p* < 0.05, ^**^ *p* < 0.01, ^***^ *p* < 0.001

To statistically understand the higher-order interaction involving time window, we conducted separate three-way ANOVAs (Age Group × Stimulation × Task Condition) for the aperiodic exponent within each time window. In the pre-trial window, only the main effect of age group was significant (*F*_[1,73]_ = 7.12, *p* = 0.009, η2 p = 0.09), with younger adults (1.06 μV/m^2^ ± 0.06) showing generally higher aperiodic exponents than older adults (0.85 μV/m^2^ ± 0.05). No other significant main effects or interactions were observed. This suggest that baseline aperiodic activity was not differentially influenced by stimulation, or task condition. In contrast, the within-trial window revealed several notable effects. A main effect of age group was significant (*F*_[1,73]_ = 6.48, *p* = 0.013, η2 p = 0.08, 95% CI [0.043, 0.356]), with younger adults exhibiting higher exponents (1.09 μV/m^2^ ± 0.05) than older adults (0.89 μV/m^2^ ± 0.06). A main effect of task condition also emerged (*F*_[1,73]_ = 48.65, *p* < 0.001, η2 p = 0.40, 95% CI [0.042, 0.069]), with Go trials (0.96 μV/m^2^ ± 0.04) eliciting lower aperiodic exponents than Nogo trials (1.00 μV/m^2^ ± 0.04). Importantly, an Age Group × Task Condition interaction was observed (*F*_[1,73]_ = 9.95, *p* = 0.002, η2 p = 0.12), the comparisons of different task conditions are shown in Fig. 4A. Simple effect analyses revealed that, during Go/Nogo trials, younger adults exhibited significantly higher aperiodic exponents than older adults (Go trials: 1.05 μV/m^2^ ± 0.05 vs. 0.87 μV/m^2^ ± 0.06, *t* = 2.28, *p* = 0.025, *d* = 0.53, 95% CI = [0.023, 0.338]; Nogo trials: 1.12 μV/m^2^ ± 0.05 vs. 0.90 μV/m^2^ ± 0.06, *t* = 2.80, *p* = 0.007, *d* = 0.65, 95% CI = [0.063, 0.376]). Furthermore, younger and older adults showed significantly higher aperiodic exponents in Nogo trials compared to Go trials during the within-trial window (younger adults: 1.12 μV/m^2^ ± 0.05 vs. 1.05 μV/m^2^ ± 0.05, *t* = 5.42, *p* < 0.001, *d* = 0.19, 95% CI = [0.045, 0.081]; older adults: 0.90 μV/m^2^ ± 0.06 vs. 0.87 μV/m^2^ ± 0.06, *t* = 4.50, *p* = 0.007, *d* = 0.07, 95% CI = [0.007, 0.041]). These results indicate that aperiodic neural dynamics are most strongly differentiated by age and task demands during active task engagement, particularly under conditions requiring greater metacontrol persistence/inhibitory control.

To test Hypothesis 1 and clarify how stimulation and task effects manifested in younger and older adults, we conducted separate two-way ANOVAs (Stimulation × Task Condition) for each age group. In the younger group, a significant main effect of task condition was found (*F*_[1, 35]_ = 29.40, *p* < 0.001, η2 p = 0.46, 95% CI [0.039, 0.087]), indicating elevated aperiodic exponents in Nogo trials (1.12 μV/m^2^ ± 0.05) relative to Go trials (1.05 μV/m^2^ ± 0.05), regardless of stimulation condition. No significant main effect of stimulation or interaction effect was observed. In contrast, the older group showed significant main effects of both stimulation (*F*_[1, 37]_ = 4.13, *p* = 0.049, η2 p = 0.10, 95% CI [0.0001, 0.104], see Fig. 4B) and task condition (*F*_[1, 37]_ = 20.25, *p* < 0.001, η2 p = 0.35, 95% CI [0.013, 0.035]). Specifically, atDCS increased aperiodic exponents (0.91 μV/m^2^ ± 0.06) compared to sham stimulation (0.86 μV/m^2^ ± 0.06), indicating that, as predicted by our first hypothesis, atDCS is a valid means to decrease neural noise in aging. Further, Nogo trials (0.90 μV/m^2^ ± 0.06) elicited higher exponents than Go trials (0.87 μV/m^2^ ± 0.06). These results suggest that stimulation-related modulation of aperiodic activity is more prominent in older adults and may reflect increased neural responsiveness under cognitive control demands.

To further examine how stimulation modulated age-related differences, we performed separate two-way ANOVAs (Age Group × Task Condition) within each stimulation condition during the within-trial window. Under sham stimulation, significant main effects were observed for age group (*F*_[1,73]_ = 7.70, *p* = 0.007, η^2^ₚ = 0.10, 95% CI [0.064, 0.392]) and task condition (*F*_[1,73]_ = 17.67, *p* < 0.001, η^2^ₚ = 0.20, 95% CI [0.023, 0.066]). Younger adults exhibited higher aperiodic exponents (1.09 μV/m^2^ ± 0.06) than older adults (0.86 μV/m^2^ ± 0.06), and Nogo trials (0.99 μV/m^2^ ± 0.04) elicited greater exponents than Go trials (0.95 μV/m^2^ ± 0.04). No significant interaction was found, indicating that task-related modulation was similar across age groups under sham conditions. In contrast, under atDCS stimulation, a significant Age Group × Task Condition interaction was observed (*F*_[1,73]_ = 15.84, *p* < 0.001, η^2^ₚ = 0.18), suggesting that stimulation enhanced differential sensitivity to task demands between age groups (see Fig. 4C for details). Simple effects analyses revealed that, during Nogo trials, younger adults (1.12 μV/m^2^ ± 0.06) showed significantly higher aperiodic exponents than older adults (0.92 μV/m^2^ ± 0.06; *t* = 2.36, *p* = 0.044, *d* = 0.55, 95% CI [0.030, 0.355]). Moreover, within each age group, Nogo trials consistently elicited greater aperiodic exponents than Go trials (younger adults: 1.12 μV/m^2^ ± 0.06 vs. 1.05 μV/m^2^ ± 0.06; *t* = 7.92, *p* < 0.001, *d* = 0.19, 95% CI [0.048, 0.078]; and older adults: 0.92 μV/m^2^ ± 0.06 vs. 0.90 μV/m^2^ ± 0.06; *t* = 3.17, *p* = 0.004, *d* = 0.06, 95% CI [0.007, 0.036]). Taken together, these findings indicate that while task-related increases in aperiodic activity are observed in both age groups, atDCS selectively enhances these effects in older adults, particularly under inhibitory control demands. This suggests a potential neuromodulatory mechanism through which atDCS may reduce cortical noise and support cognitive control processes that are sensitive to aging.

### Correlations: analysis to test Hypothesis 2

Analysis of the above aperiodic exponent supported our first confirmatory hypothesis that atDCS significantly enhances aperiodic exponents in older adults, particularly during Nogo trials, indicating reduced cortical noise under stimulation. To quantify this neuromodulatory effect, we calculated ΔFOOOF-atDCS (i.e., atDCS FOOOF effect minus sham FOOOF effect), reflecting the individual-specific magnitude of aperiodic activity modulation induced by atDCS. To test the second confirmatory hypothesis—that baseline aperiodic activity predicts the extent of neuromodulatory response to atDCS in both younger and older adults—we conducted correlation analyses between the resting-state aperiodic exponent and ΔFOOOF-atDCS. Contrary to our expectations, the results revealed no significant association between resting-state aperiodic index and ΔFOOOF-atDCS magnitudes in either the younger group (*r* = 0.13, *p* = 0.443) or the older group (*r* = −0.12, *p* = 0.481). These findings imply that baseline aperiodic activity is not a reliable predictor for task-related stimulation effects induced by atDCS across age groups. This indicates that other neurobiological factors—such as cortical thickness or structural integrity—may play a more relevant role in explaining individual differences in responsivity to atDCS.

Exploratory analyses found no evidence that atDCS modulated task-related theta or alpha power (Supplementary Material S2.1). Furthermore, an analysis investigating the relationship between atDCS-induced changes in the aperiodic exponent and oscillatory power also revealed no significant correlations (S2.2). This dissociation suggests that, under the present conditions, the periodic and aperiodic components of the EEG signal represent distinct and independent neurophysiological responses to neuromodulation.

## Discussion

The goal of this study was to investigate whether age-related alterations in metacontrol—the ability to adaptively adjust cognitive control to task demands—can be modulated by noninvasive brain stimulation and are reflected in aperiodic and periodic neural activities. Specifically, we examined whether atDCS can modulate the GABAergic inhibitory tone and the related E/I balance [29, 30], and whether such effects differ between younger and older adults. To capture neural signatures of metacontrol, we analyzed aperiodic activity using the FOOOF algorithm, which parameterizes the EEG power spectrum into periodic and aperiodic components [14]. Critically, we interpret an "elevated spectral slope" as an increase in the aperiodic exponent. While FOOOF exponents can be positive or negative, a higher value corresponds to a steeper slope in log–log space, conventionally reflecting stronger inhibitory regulation and more stable neural dynamics [17, 18, 20, 21, 34, 35]. Complementing this approach, we examined periodic activity through time–frequency analysis of theta and alpha oscillations, frequency bands strongly implicated in cognitive control and inhibition [27, 28]. Building upon this theoretical framework, we formulated three hypotheses: (1) that atDCS, compared to sham stimulation, would modulate the inhibitory tone during cognitive task performance, particularly in older adults; (2) that baseline resting-state aperiodic activity would predict the magnitude of neuromodulatory response to atDCS in both age groups; and (3) that aperiodic and periodic (i.e., theta, alpha) neural activities would exhibit covariation under atDCS stimulation. To address these questions, we implemented a mixed design wherein younger and older participants received both active and sham stimulation while performing a Go/No-Go task—a well-established paradigm for eliciting differential control demands and assessing metacontrol dynamics. Our findings provide partial support for the first hypothesis while indicating that the latter two hypotheses were not substantiated by the data, thereby offering refined insights into the electrophysiological markers underlying age-related metacontrol and their responsiveness to neuromodulation.

The first hypothesis suggested that atDCS, compared to sham stimulation, would reduce cortical neural noise—reflected by an increase in the aperiodic exponent derived from spectral parameterization—particularly in older adults. This prediction is partially supported by our data. The critical finding that no stimulation effects were present in the pre-trial baseline definitively demonstrates that atDCS does not exert a global effect on the baseline aperiodic slope. Instead, its neuromodulatory action is state-dependent, specifically enhancing the brain's dynamic task-response ability during active engagement. Most importantly for Hypothesis 1, the older group exhibited a significant stimulation effect in the within-trial interval, where atDCS increased the aperiodic exponent relative to sham. Furthermore, the significant Age Group × Task Condition interaction observed under atDCS indicates that this facilitatory effect is further modulated by inhibitory demand, enhancing neural differentiation in both age groups under high-control conditions. Together these results indicate that atDCS can enhance cortical signal fidelity in aging, but that this neuromodulation is not uniform — it is temporally constrained (expressed during task engagement), task-sensitive (stronger under high inhibitory demand), and interacts with age differences (younger participants still showed relatively larger exponents in high-demand condition). Functionally, this pattern is consistent with the view that atDCS shifts local E/I balance toward greater inhibition/improved SNR [24, 35], yet the expression of that shift depends on ongoing cognitive demands and the ageing brain’s starting point. In other words, atDCS appears to reduce spontaneous background activity most effectively when participants are actively engaged in persistence/inhibitory control, and in older adults this produces measurable increases in the exponent even if it does not fully normalize values to the younger profile. These conditional effects underscore that the neurophysiological index of atDCS efficacy is not a static baseline measure, but rather a dynamic modulation of neural responsiveness during task performance.

The second hypothesis proposed that individual differences in baseline aperiodic activity during resting-state EEG would predict the degree of aperiodic modulation induced by atDCS, with the assumption that both younger and older adults—particularly older adults with lower inhibitory tone (i.e., lower exponents)—would benefit more from stimulation. However, contrary to our expectations, correlation analysis revealed no significant relationship between resting-state aperiodic exponents and ΔFOOOF-atDCS in either younger or older adults. Several potential explanations may account for this null finding. First, it is possible that the neuromodulatory effects of atDCS do not linearly scale with baseline neural noise levels in each age group, where neurobiological factors such as cortical atrophy, white matter integrity, or neurotransmitter system alterations could modulate responsiveness to stimulation [25, 26, 28]. Second, while aperiodic activity in resting-state EEG has been proposed as a trait-like marker of E/I balance [17], its predictive validity for stimulation effects occurring during active task engagement may be limited due to the mismatch in cognitive context and neural state. In other words, the lack of temporal and contextual alignment between resting-state baseline and task-specific activity may weaken the sensitivity of this predictor [35]. Additionally, individual variability in atDCS efficacy is a well-documented phenomenon and may arise from anatomical factors (e.g., skull thickness, cortical folding), functional brain states, and even genetic variability affecting neuroplasticity [25, 26]. Furthermore, Liu et al. demonstrated in a motor imagery-based brain–computer interface (BCI) paradigm that transcranial alternating current stimulation (tACS) could enhance task-specific neural signals (µ ERDs) and improve online BCI performance, but critically, the efficacy was contingent on individualized peak resting-state sensorimotor rhythms [36]. This mechanistic link between resting-state spectral features and task-related modulation supports our interpretation that state-dependent stimulation effects are complex and cannot be fully predicted by resting-state metrics alone.

It is also possible that aperiodic EEG metrics alone are insufficient to fully capture the complexity of metacontrol-related modulation. A more comprehensive prediction model might integrate structural imaging (e.g., MRI), neurotransmitter mapping (e.g., MRS), and behavioral traits to stratify participants by stimulation susceptibility. Future research should therefore combine multimodal neuroimaging and personalized modeling approaches to better elucidate the mechanisms driving inter-individual differences in atDCS outcomes.

In contrast to the robust modulation observed in aperiodic activity, our analyses of periodic oscillatory power revealed no significant effects of atDCS stimulation. Neither the electrode-specific analysis at Cz nor the exploratory whole-scalp cluster-based permutation tests detected reliable stimulation-related changes in theta or alpha band power in either age group. This null effect was observed despite clear task-related modulations—specifically, enhanced theta and alpha power during Nogo compared to Go trials across both younger and older adults. The dissociation between aperiodic and periodic outcomes suggests that the neuromodulatory influence of atDCS under these experimental conditions may be more strongly expressed in the broadband aperiodic component of the EEG signal, rather than in narrowband oscillatory dynamics. Furthermore, the absence of any correlation between stimulation-induced changes in the aperiodic exponent and theta power (ΔFOOOF-atDCS and Δtheta-atDCS) reinforces the view that these two neurophysiological signatures may reflect distinct mechanisms of cortical activity, with atDCS primarily affecting the non-oscillatory, scale-free neural background. Future research employing different stimulation parameters, longer durations, or task designs with higher cognitive demand may be needed to elicit reliable oscillatory modulation.

We found no effect of atDCS on behavioral markers of metacontrol, such as accuracy or response time in the Go/Nogo task. Younger adults exhibited higher accuracy and faster responses than older adults in the Go condition, but stimulation did not significantly influence performance in either age group, nor were there any stimulation × group interaction effects. This pattern aligns with previous studies showing that tDCS often fails to elicit measurable behavioral improvements across various cognitive domains, including working memory and task switching [37, 38]. It is noteworthy that while several studies have established a link between the resting-state aperiodic slope and cognitive performance in older adults—particularly in domains such as information processing speed and global cognitive function [39, 40]—our study focused on task-evoked, state-dependent neural dynamics. This distinction may be critical: the predictive relationship observed at rest may not directly translate to state-specific neural changes elicited during task performance, especially within the context of a targeted neuromodulation intervention. Notably, several of these studies nonetheless observed neural changes, such as altered EEG power or functional connectivity, despite the absence of overt behavioral benefits. This discrepancy highlights a critical insight: the lack of behavioral effects does not necessarily indicate that atDCS was ineffective. While it is possible that atDCS engages latent neural mechanisms without immediate behavioral manifestation, an equally plausible explanation is that the specific neural processes modulated by atDCS—as indexed by the aperiodic exponent—may not be the primary limiting factor for performance in this particular task context. Consequently, the absence of a behavioral effect tempers strong claims regarding the immediate practical utility of atDCS for enhancing cognition in aging. Instead, our findings highlight the value of neural markers such as the aperiodic exponent [14] as sensitive indicators of metacontrol adjustments, which may precede or operate independently of overt behavioral changes. Future research should investigate whether these atDCS-induced neural adjustments translate to behavioral benefits in more complex or ecologically valid scenarios where cognitive resources are more substantially challenged.

In sum, the present study provides preliminary evidence that anodal tDCS can modulate aperiodic neural activity, with these effects emerging in an age- and task-specific manner. Specifically, atDCS enhanced the aperiodic exponent—suggesting a strengthening of inhibitory tone—in older adults during task performance. Notably, these neurophysiological effects emerged without concomitant behavioral improvements, indicating a dissociation between neural modulation and overt performance in this task context. Furthermore, the absence of significant stimulation effects on oscillatory power and the lack of correlation between aperiodic and periodic components suggest that these neural signatures may represent distinct mechanisms of cortical activity. Together, these findings highlight the value of aperiodic measures as sensitive indicators of neuromodulatory effects in the aging brain, while underscoring the complexity of translating neurophysiological changes into behavioral benefits.

Several limitations of the current study should be considered. First, our sample size, while sufficient to detect the primary neurophysiological effects, may have limited power for exploring more nuanced individual differences or specific subgroup analyses. Second, an interesting observation emerging from our spectral data, particularly in the older adult group, is the potential presence of a bimodal scaling regime. This phenomenon, previously reported in aging populations [41, 42], suggests that the electrophysiological processes governing low-frequency and high-frequency neural activity may become more dissociated with age. While our study employed a standard FOOOF model with a single aperiodic exponent across the 3–40 Hz range for consistency, we acknowledge that this approach may average over these distinct scaling regimes. Future investigations specifically designed to model such spectral breakpoints could yield more nuanced insights into the differential effects of aging and neuromodulation. Finally, although we assessed basic sensory experiences and employed a single-blind design, future studies would benefit from more rigorous blinding procedures and comprehensive assessment of participant-level factors that may influence stimulation efficacy.

Our results have intriguing implications for the realm of aging research. While aging is often linked to increased cortical neural noise [43], which can impair cognitive persistence [17–21], our results suggest that non-invasive neuromodulation can attenuate neural noise and selectively enhance metacontrol-related oscillatory activity under high control demands, particularly in older adults. The absence of comparable effects in younger adults may reflect a ceiling effect, as their neural systems are already highly efficient and less susceptible to further modulation. These preliminary yet promising findings contribute to the broader discussion on neuroplasticity in aging, suggesting that even later in life, the brain retains a degree of plastic capacity. Future research should therefore focus on refining stimulation protocols, identifying reliable biomarkers of responsivity, and determining whether the observed neural benefits can translate into functional cognitive gains in daily life. Targeting metacontrol mechanisms through neuromodulation may, with further validation, open new avenues for supporting cognitive health in older adults.

## Supplementary Information

Below is the link to the electronic supplementary material.

Supplementary file1 (DOCX 26 KB)

## Data Availability

The aggregated data analyzed in this article are available via the Open Science Framework: https://osf.io/7cj8k/. All data can be obtained from Yu Pi (963445475@qq.com) upon reasonable request.
