# Supplementary material for: Metacontrol-related aperiodic and periodic neural activity in cognitive aging: enhancing the neural signal-to-noise ratio through anodal transcranial direct current stimulation
Source: GeroScience. 2026 Jan 16;48(6):7887–903. doi: 10.1007/s11357-025-02077-8 (PMC13638896; doi:10.1007/s11357-025-02077-8)
Supplement: Supplementary file 1 [file 11357_2025_2077_MOESM1_ESM.docx]

**Supplementary Materials**

**Metacontrol-related aperiodic and periodic neural activity in aging: evidence from anodal transcranial direct current stimulation**

Yu Pi, Qinfei Zhang, Shuhui Lyu, Christian Beste, Lorenza Colzato, Bernhard Hommel

# Supplementary Methods

# *S1.1 Parameterization of the spectral data*

The FOOOF algorithm facilitates the decomposition of the signal into an aperiodic component (characterized by a 1/f slope) and a periodic component (oscillations), enabling the parametrization of the power spectrum [28]. The power spectrum model is articulated as:

$$PSD\left( f \right)=L\left( f \right)+\sum_{n} G_{n}\left( f \right)$$

Here, $L\left( f \right)$ denotes the aperiodic component, and $\sum_{n} G_{n}\left( f \right)$ signifies the periodic component, with f representing frequency. The model essentially portrays the power spectrum as a linear combination of the aperiodic component, $L\left( f \right)$, and a series of n Gaussian functions. The aperiodic component is modeled as a function that extends across the entire spectral range and is defined by two parameters. The expression for the aperiodic component function $L\left( f \right)$ is:

$$L\left( f \right)=b-\log[f^{x}]$$

In this equation, b corresponds to the overall 'offset' of the aperiodic broadband power, x denotes the 'exponent' of the aperiodic fit, reflecting the slope of the power spectral line on a log-log scale, and f indicates the frequency of the aperiodic exponent. Periodic components, also referred to as oscillatory components, relate to specific frequency bands where power surpasses that of the aperiodic component. Each oscillatory component, or "peak," is represented by a Gaussian model and is defined by three parameters. The periodic component function $G_{n}\left( f \right)$ is expressed as:

$$G_{n}\left( f \right)=a_{n}\exp[-\frac{\left( f-\mu_{n} \right)^{2}}{2\sigma_{n}^{2}}]$$

where $a_{n}$ represents the amplitude, $\mu_{n}$ the center frequency, and $\sigma_{n}$ the bandwidth. Following this, a frequency range of 4 to 40 Hz was selected for fitting each spectrum, with parameters set as {peak_width_limits = [2, 8], min_peak_height = 0.05, max_n_peaks = 8, aperiodic_mode = 'fixed'}. Power spectra were fitted for every electrode, participant, task condition, and period to derive an aperiodic exponent value. The spectral fit's average R² across the 75 participants was 0.92, signifying a good fit.

# *S1.2 Time-frequency analysis*

To examine induced (non-phase-locked) oscillatory activity in the theta and alpha bands during the Go/Nogo task, time-frequency (TF) decomposition was applied following a procedure to isolate non-phase-locked components. Specifically, to minimize contamination from phase-locked evoked potentials, we first computed the event-related potential (ERP) for each participant and condition by averaging across all trials. This ERP was then subtracted from each individual trial prior to time-frequency analysis, ensuring that the resulting spectral estimates reflect primarily induced (non-phase-locked) oscillatory activity. The TF analyses employed a continuous wavelet transformation using Morlet wavelets (w), defined as:

$$w\left( t,f \right)=Aexp\left( -t^{2}/ \right.\left. {2\sigma}_{t}^{2} \right)exp\left( 2i\pi ft \right)$$

where $t$ represents time, $f$ represents frequency, $A$ = $\left( \sigma_{t} \right.{\sqrt{\pi})}^{-1/2}$, $\sigma_{t}$ represents wavelet duration, and i represents $\sqrt{-1}$. A Morlet parameter of $f_{0}/\sigma_{f}$ = 7 was used, with $f_{0}$ denoting the central frequency and $\sigma_{f}$ defining the Gaussian shape in the frequency domain. Wavelet duration ${2\sigma}_{t}$ and spectral bandwidth ${2\sigma}_{f}$ are functions of $f_{0}$, and the relationship between $\sigma_{t}$ and $\sigma_{f}$ is given by $\sigma_{t}$ = $1/\left( 2\pi\sigma_{f} \right)$. TF decomposition was performed across a frequency range of 3–40 Hz in 38 steps, and over a time window from -0.5 to 1.5 seconds relative to stimulus onset. To normalize power values, baseline correction was applied using the pre-stimulus interval from -0.5 to -0.1 seconds. Following the ERP-subtraction procedure and time-frequency decomposition, TF power was averaged across trials for each participant separately. To identify spatiotemporal regions showing prominent task-related power changes, grand-average time–frequency representations (TFRs) across all channels were first visualized for each of the four experimental conditions using the ft_multiplotTFR function. This visual inspection revealed that the most pronounced power increases in the theta band (3–7 Hz) and the alpha band (9–12 Hz) occurred at electrode Cz. Following visual inspection of the grand average scalp topography for each condition: 1) the theta band was defined as the time window of 0.2-0.6 s and the frequency range of 3-7 Hz; 2) Alpha band activity was defined as the time window of 0.3–0.7 s and the frequency range of 9–12 Hz. The average power within these time-frequency windows was extracted across channels for the theta and alpha bands, followed by a repeated-measures ANOVA on the resulting power values. To further explore potential effects across the entire sensor space, we conducted a nonparametric cluster-based permutation test using the MNE-Python toolbox. This test compared total theta power between Go and NoGo trials under atDCS versus sham conditions across multiple channels, separately for theta and alpha bands. Clusters were defined as spatiotemporal samples where the F-value exceeded a significance threshold of p < 0.05, requiring at least two EEG channels to define a cluster. The reference distribution for the permutation test was approximated using a Monte Carlo method with 1,000 random permutations. Clusters with corresponding *p*-values below the critical alpha level of *p* < 0.05 were considered statistically significant. For significant clusters, the participants' mean total power during this time–frequency range (theta: 0.2–0.6 s, 3–7 Hz; alpha: 0.3–0.7 s, 9–12 Hz) for Go and NoGo trials in the atDCS/sham stimulations was used for subsequent statistical analysis.

*S1.3 Statistical analysis*

For the statistical analysis of time-frequency data, we employed a hierarchical analytical approach. First, a three-way mixed-design ANOVA was conducted with Group (younger vs. older) as the between-subjects factor, and Stimulation (atDCS vs. sham) and Task (Go vs. NoGo) as within-subjects factors. Subsequently, to examine stimulation effects within each age group, separate two-way repeated-measures ANOVAs (Stimulation × Task) were performed for younger and older adults separately. If no significant effects were observed at the selected electrodes, an exploratory analysis was conducted using MNE-Python’s nonparametric cluster-based permutation test on the full scalp time-frequency data to identify significant clusters exhibiting differences in theta total power under the 2 (Stimulation) × 2 (Go/Nogo) conditions. Power values from electrodes contributing to the significant cluster were averaged, and the resulting data were subjected to a repeated-measures ANOVA with stimulation and Go/Nogo as within-participant factors, restricted to the within-trial time window. For the alpha band, the same analysis procedure as the theta band was applied.

Finally, to test the explorative third hypothesis of an interrelation between stimulation-induced changes in aperiodic activity and oscillatory dynamics triggered by atDCS in each age group, we computed Δtheta-atDCS (i.e., theta total power difference between atDCS and sham conditions) and examined its correlation with ΔFOOOF-atDCS (i.e., difference in FOOOF exponent between atDCS and sham) across participants. To ensure spatial and temporal alignment between these two measures, the ΔFOOOF-atDCS value was extracted using the same time window (100–600 ms) and same electrodes as used in the Δtheta-atDCS calculation. This spatial and temporal alignment was adopted to control for potential confounds due to differences in signal origin and dynamics, thereby allowing a more direct comparison of modulation effects across spectral components.

# Supplementary Results

*S2.1* *Theta and alpha results*

To investigate potential modulation of oscillatory power by age, stimulation, and task condition, we first conducted a three-way repeated-measures ANOVA for the theta band with Group (younger, older) as a between-subjects factor, and Stimulation (atDCS, sham) and Task Condition (Go, NoGo) as within-subjects factors. Results revealed significant main effects of Group and Task Condition, as well as a significant Group × Task Condition interaction. Simple effects analyses showed that younger adults exhibited higher theta power than older adults specifically in the NoGo condition, whereas both groups demonstrated significantly greater theta power in NoGo compared to Go trials. To further explore stimulation effects, we conducted separate two-way ANOVAs (Stimulation × Task Condition) within each age group. These analyses consistently revealed a significant main effect of Task Condition (NoGo > Go) in both groups, but no significant main effect of Stimulation or interaction between Stimulation and Task Condition. We then performed a parallel three-way repeated-measures ANOVA for the alpha band. Results revealed only a significant main effect of Task Condition, but no other significant main effect or interactions. Follow-up two-way ANOVAs within each age group again showed a significant Go/Nogo effect (NoGo trials > Go trials), with no significant effects of Stimulation or interaction terms. Notably, this pattern closely resembled the theta findings, suggesting that task-related modulation of oscillatory power was robust across frequency bands, while tDCS had no reliable influence on oscillatory dynamics at the Cz electrode, indicating that average power at this manually selected site did not differ significantly across stimulation conditions.

To complement these electrode-specific analyses and examine potential distributed neural effects, we conducted exploratory whole-scalp cluster-based permutation tests. Critically, these analyses identified no significant clusters exhibiting a stimulation × task condition interaction in either the theta or alpha bands for both age groups. This pattern of null findings across both frequency bands and analytical approaches indicates that while task-related modulation of oscillatory power was robust, tDCS stimulation did not produce reliable effects on oscillatory dynamics at either the predefined Cz electrode or across the broader scalp topography.

*S2.2 Correlations FOOOF and oscillatory dynamics: exploratory analysis to test Hypothesis 3*

Given the absence of significant stimulation × task condition interactions in the theta and alpha bands from our primary analyses, we conducted an exploratory correlation analysis to examine the potential relationship between stimulation-induced changes in aperiodic and oscillatory neural dynamics. This analysis was motivated by the theoretical possibility that these two neural signatures might co-vary in response to neuromodulation, even in the absence of overall group-level stimulation effects. We computed change scores for both aperiodic exponent (ΔFOOOF-atDCS) and theta power (Δtheta-atDCS) by subtracting sham values from atDCS values across all participants. To ensure spatial consistency and maximize signal-to-noise ratio, both measures were derived from global averages across all electrodes.

A Pearson correlation analysis revealed no significant associations between stimulation-induced changes in aperiodic and oscillatory activity: ΔFOOOF-atDCS was not significantly correlated with Δtheta-atDCS (r = -0.06, p = 0.604) nor with Δalpha-atDCS (r = 0.08, p = 0.517) across the entire sample. These consistent null findings suggest that, under the current experimental conditions, changes in aperiodic and periodic neural activity in response to atDCS represent largely independent neurophysiological processes. The dissociation between these neural signatures across both frequency bands indicates that they may reflect distinct mechanisms of neuromodulation, potentially involving different aspects of cortical excitability and network dynamics.
